# Enteroaggregative and Enteroinvasive Escherichia coli as a Cause of Pediatric Acute Abdomen: A Report of Two Cases

**DOI:** 10.7759/cureus.68340

**Published:** 2024-08-31

**Authors:** Natalie Schneeweiss Garber, Paul A Bourgade Su, Gretel Lozano Guerrero, Andrea Hernandez Salazar, Carlos Manuel Aboitiz

**Affiliations:** 1 Faculty of Health Sciences, Universidad Anáhuac México, Mexico City, MEX; 2 Pediatrics, Hospital Angeles Pedregal, Mexico City, MEX; 3 Pediatric Cardiology, Hospital Angeles, Mexico City, MEX; 4 Cardiology, Instituto Nacional de Enfermedades Respiratorias, Mexico City, MEX

**Keywords:** case report pediatric, enteroinvasive escherichia coli, enteroaggregative escherichia coli, acute abdomen, abdominal pain

## Abstract

Abdominal pain stands as one of the foremost reasons for consultation among pediatric patients, presenting a diagnostic challenge owing to its diverse underlying causes. The manifestation of abdominal pain varies according to age, associated symptoms, and pain localization. While frequently self-limited, certain conditions exist that endanger life and require urgent intervention. Acute abdomen denotes severe, non-traumatic abdominal pain resulting from inflammatory, ischemic, obstructive, infectious, gynecological, or metabolic etiologies, warranting immediate therapeutic intervention.

Infectious processes that mimic acute abdominal conditions are relatively uncommon. Consequently, the identification of infectious gastroenteritis as a probable etiology of acute abdomen can prevent unnecessary surgical interventions in patients. This report details two cases: a 14-year-old pediatric patient presenting with acute abdominal pain, in whom appendiceal involvement was ruled out through ultrasonographic and computed tomography (CT) examinations, confirming the presence of enteroaggregative *Escherichia coli*, and a 10-year-old pediatric patient presenting with a sudden onset of abdominal pain. CT findings revealed an appendiceal fecalith without concurrent inflammation but accompanied by mesenteric adenitis. Even though conservative treatment did not improve the pain, it was later determined that the patient was a carrier of enteroinvasive *E. coli*. In both cases, antimicrobial treatment with rifaximin 200 mg every eight hours was administered, leading to the resolution of the conditions without the need for hospital readmission or additional therapy.

Infectious conditions stemming from enteroaggregative and enteroinvasive *E. coli* can mimic acute abdomen and should be regarded as potential infectious etiologies when other more common causes have been ruled out.

## Introduction

It has been described that strains of *Escherichia coli* causing diarrhea in humans are classified into five pathotypes: enteropathogenic *E. coli* (EPEC), enterotoxigenic* E. coli* (ETEC), enteroinvasive* E. coli* (EIEC), enteroaggregative *E. coli* (EAEC), and Shiga toxin/verotoxin-producing or enterohemorrhagic *E. coli *(STEC/VTEC/EHEC) [1,2].

The five pathotypes of diarrheagenic *E. coli* exhibit distinct virulence mechanisms targeting enterocytes. EIEC has the ability to penetrate the body via M cells and induce apoptosis in macrophages. It subsequently invades adjacent enterocytes through the basal membrane and replicates intracellularly while evading destruction within the phagosome. Thereafter, it disseminates laterally to other enterocytes and secretes Shiga toxins. EHEC adheres via bundle-forming pili (Bfp) and secretes Shiga toxin, which is subsequently internalized through endocytosis and released into the systemic circulation. ETEC secretes heat-stable toxin (ST) and heat-labile toxin (LT), which subsequently lead to the production of cyclic AMP (cAMP) and cyclic GMP (cGMP). EAEC adheres to the cilia of enterocytes, secreting cytotoxins and inducing mucus secretion. Initially, EPEC adheres to host cells; subsequently, employing a type III secretion system, it translocates bacterial toxins. This process results in the formation of pedestals and a close attachment to the host cells [3,4].

The infectious agents associated with the high morbidity and mortality of acute diarrheal disease (ADD) include viruses, bacteria, and, to a lesser extent, parasites. Among the viral causes of ADD, rotavirus is the most significant, associated with approximately 440,000 annual deaths, 82% of which occur in the world's poorest countries [5]. Bacterial causes of ADD rank second in frequency, with enteropathogenic strains of *E. coli* being the most important. *E. coli* strains are significant causes of ADD in children under five years old, particularly in Latin America, Africa, and Asia, and are associated with high mortality rates. The literature, studies, and epidemiological reports on intestinal pathogens causing ADD in Latin America are limited due to the lack of detection tests for these pathogens in public health centers [5].

EAEC is an emerging type of *E. coli* that has been identified as a causative agent of traveler's diarrhea, gastroenteritis in children residing in developing countries, especially in cases of persistent diarrhea lasting over 14 days, and chronic diarrhea in individuals with human immunodeficiency virus (HIV) [6,7]. It stands as one of the primary contributors to morbidity and mortality in this context. Furthermore, it has been associated with a foodborne pathogen responsible for sporadic diarrhea in healthy adults and children in developed countries [6]. Its pathogenesis involves a proposed model in which the bacteria produce enterotoxins and mucosa-adhering cytotoxins, leading to a self-limiting watery diarrhea lacking mucus or blood, while concurrently inflicting damage to the intestinal mucosa [1,6-8].

On the other hand, EIEC has been identified as an invasive strain causing dysentery. It has been reported endemically in developing countries, accounting for a frequency of 1-5% of diarrhea episodes in the population. Its mechanism of pathogenicity involves the production of enterotoxins and mucosa-adhering cytotoxins, followed by the colonization and invasion of colonic cells, subsequently leading to their destruction, and triggering an inflammatory process. This cascade results in self-limiting watery diarrhea, accompanied by the presence of mucus and/or blood [9].

It is uncommon for infectious processes to manifest as acute abdomen symptoms. Infections caused by EAEC and EIEC typically manifest with watery diarrhea rather than severe abdominal pain [6,7,10]. Abdominal pain is reported as an infrequent symptom, though, when present, it can become the prominent symptom, mimicking an acute abdomen presentation [7,11].

Cases of acute abdomen attributable to infectious processes are notably rare, and there is a lack of specific data in the literature due to their infrequent incidence. Consequently, these cases warrant particular attention in order to avoid and prevent unnecessary surgical procedures [7]. Gastroenteritis represents the most common non-surgical etiology of abdominal pain, whereas appendicitis is recognized as the most prevalent surgical condition [11].

In this article, we present two cases involving the challenging diagnosis of acute abdomen scenarios resulting from infections caused by EAEC and EIEC. 

## Case presentation

Case 1

A 14-year-old, female patient, with no significant medical history, presented to the emergency department with a two-day history of abdominal pain, nausea, and reduced appetite. The patient was afebrile and had no history of vomiting or chills. Upon questioning, she described acute abdominal pain initially localized in the mesogastrium that migrated to the right iliac fossa, with an intensity of 8/10. The pain was constant, radiating to the back, and had a sudden onset. Abdominal distension and loose stools were also observed, with no mucus or blood. The patient and her family denied recent travel or consumption of food outside their home. 

**Figure 1:**
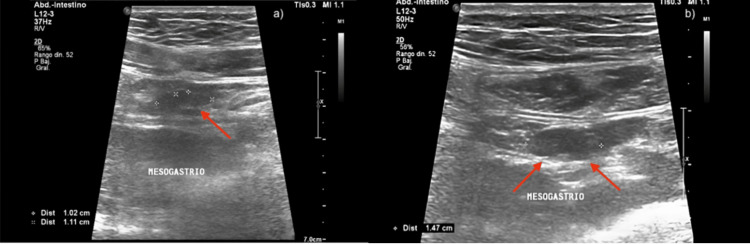
Abdominal ultrasound. (a) Several lymph nodes with swollen appearance are observed. (b) A swollen lymph node measuring 14 mm, suggestive of mesenteric adenitis, and a small amount of free fluid in the right iliac fossa are noted.

**Figure 2:**
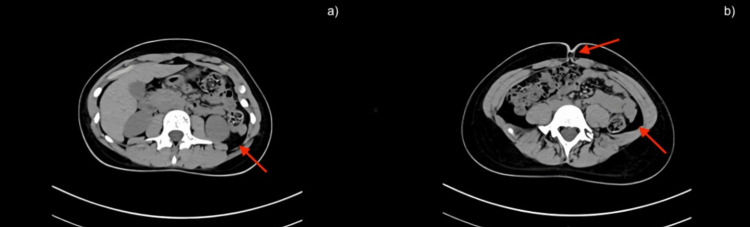
Axial abdominal CT scan. (a, b) Free fluid is observed in the posterior cul-de-sac. Small umbilical hernia. Findings consistent with mesenteric adenitis. CT: computed tomography

Upon admission, the patient presented with stable vital signs. Physical examination revealed abdominal tenderness, predominantly localized in the right iliac fossa, accompanied by positive signs such as Blumberg's sign, McBurney's sign, and also a positive psoas sign. Percussion elicited generalized tympanic resonance and decreased peristalsis, while no visceromegaly was observed. The rest of the physical examination yielded no relevant data. An initial abdominal X-ray revealed nonspecific findings, leading to the decision to perform an abdominal ultrasound (Figure 1), which identified mesenteric adenitis, free fluid in the abdominal cavity, and an indistinctly visualized appendix without any signs of inflammation. An abdominal computed tomography (CT) scan was also performed (Figure 2), confirming mesenteric adenitis without significant alterations.

Management was initiated with intravenous fluids, fasting, and analgesia. The following laboratory tests were conducted: complete blood count revealing a hemoglobin level of 16.1 g/dL, hematocrit of 46.6%, platelet count of 257,000/µL, white blood cell count of 8,000/µL, neutrophils at 46% (3,680/µL), lymphocytes at 47% (3,760/µL), and monocytes at 4% (320/µL). Urinalysis showed a pH of 6.5 and density of 1.005, and no other abnormalities were detected.

After 24 hours from the initial evaluation, fasting was interrupted, but food was poorly tolerated, resulting in nausea and a vomiting episode. The patient experienced increased pain in the right iliac fossa, reaching an intensity of 10/10 similar to the pain characteristics noted previously. The patient also reported decreased fecal firmness. As a result, a fecal sample was collected to conduct a gastrointestinal pathogens panel using polymerase chain reaction (PCR), which yielded a positive result for EAEC. 

Antimicrobial treatment was initiated with rifaximin 200 mg every eight hours for three days. Following this, the patient showed improvement, characterized by a gradual reduction and eventual complete resolution of abdominal pain, along with improved tolerance and acceptance of oral intake after three days of antibiotic treatment. Subsequent hematological analysis during follow-up revealed a hemoglobin concentration of 14.7 g/dL, a hematocrit value of 44%, a platelet count of 245,000/µL, a total leukocyte count of 6,700/µL, neutrophils accounting for 53% (3,550/µL), lymphocytes at 41% (2,750/µL), and monocytes at 4% (270/µL). Due to the observed clinical improvement, the patient was discharged home and continued to exhibit signs of favorable recovery, without the need for readmission or additional treatment.

Case 2

A 10-year-old male patient presented with a sudden and severe episode of abdominal pain, prompting him to seek outpatient medical attention. Upon presentation, the patient was afebrile and did not report any history of vomiting. Further inquiry only revealed that the patient had recently experienced an acute respiratory episode marked by nasal congestion. Additionally, the patient described acute pain predominantly localized in the right iliac fossa, increasing in intensity. Notably, there were no associated symptoms reported. Both the patient and his family denied recent travel or consumption of take-out food or visiting any restaurants. 

**Figure 3:**
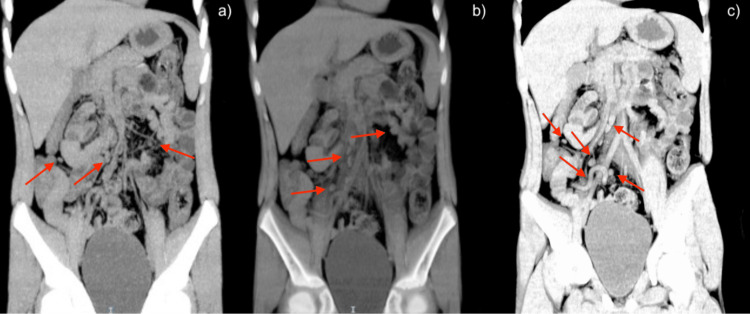
Coronally sectioned abdominal CT scan. (a, b) Findings consistent with mesenteric adenitis. Multiple oval-shaped images correspond to swollen mesenteric lymph nodes. More than five mesenteric lymph nodes are observed with short axes exceeding 10 mm. (c) Retrocecal appendix of increased density, with a maximum transverse diameter up to 4.4 mm at the base, without peripheral inflammatory changes, and with the presence of a small appendicolith at the tip measuring more than 2 mm in diameter. CT: computed tomography

**Figure 4:**
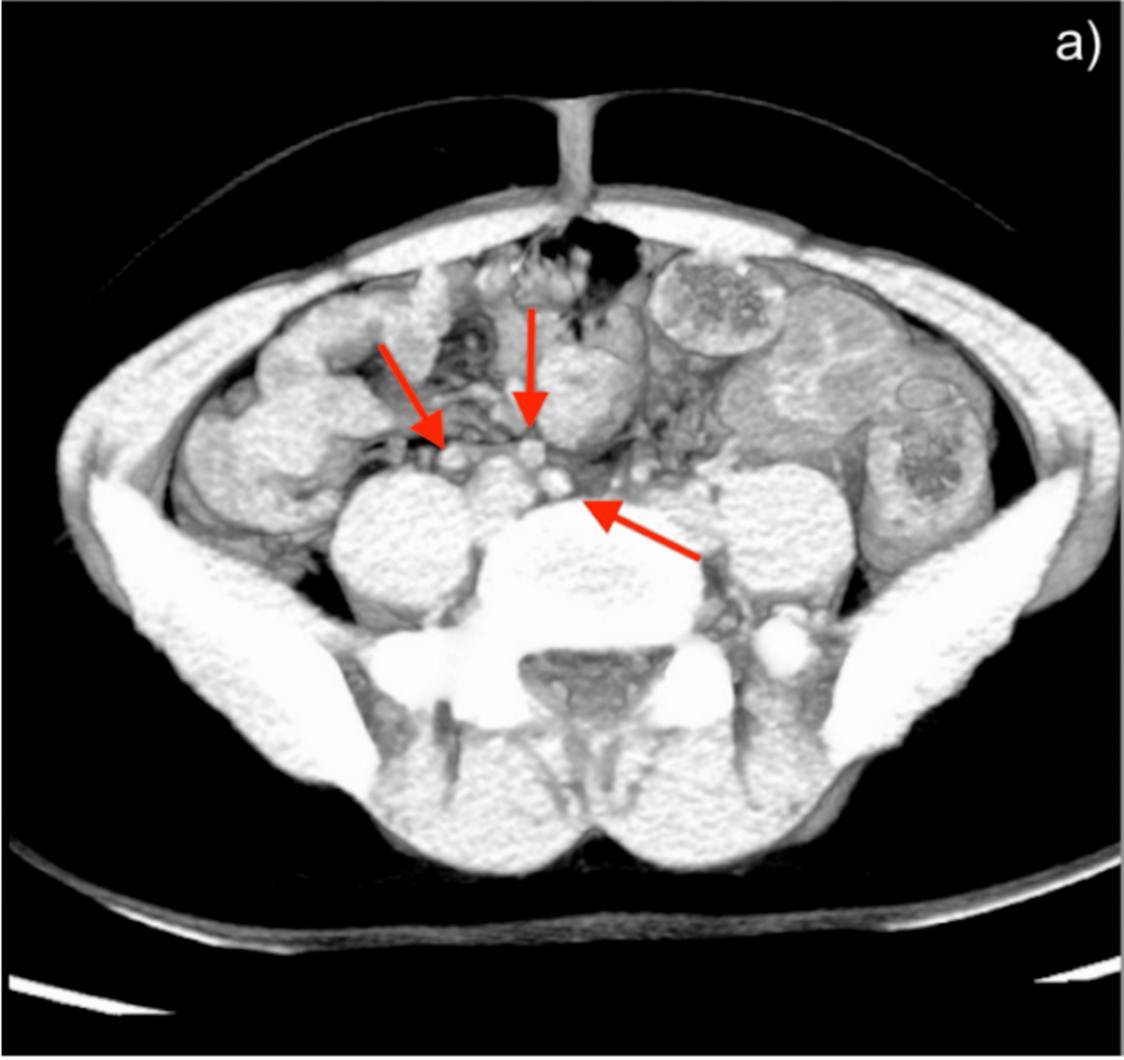
Axial abdominal CT scan. (a) Findings consistent with mesenteric adenitis. Multiple oval-shaped images correspond to swollen mesenteric lymph nodes. CT: computed tomography

Upon admission, vital signs were within normal ranges. Upper airway examination revealed a hyperemic pharynx without exudate. Clinically, there were no signs of peritoneal irritation, with the patient experiencing abdominal tenderness solely upon superficial and deep palpation in the right iliac fossa. The remainder of the physical examination was unremarkable and did not yield any pertinent findings. Due to the escalating intensity in pain, an abdominal CT scan (Figure 3 and Figure 4) was performed, which identified a retrocecal appendix with an appendicolith present but without signs of inflammation. Multiple peritoneal lymph nodes were also detected, leading to the diagnosis of mesenteric adenitis and ruling out acute appendicitis. Surgical intervention was deemed unnecessary. 

Based on the imaging findings, an inflammatory process involving the appendix was excluded, thereby eliminating the need for surgical intervention and confirming the diagnosis of mesenteric adenitis. Expectant management was initiated, including analgesia to manage pain effectively. However, due to an inadequate response and persistent moderate-intensity pain, a fecal sample was collected for a gastrointestinal pathogens panel using PCR, which returned a positive for EIEC.

Antimicrobial therapy was initiated with rifaximin 200 mg every eight hours for three days. Following this treatment, the patient showed improvement, evidenced by a reduction in abdominal pain that ultimately resolved completely. The patient exhibited good tolerance and compliance with oral food intake after three days of antibiotic treatment. Due to the marked clinical improvement, the patient was discharged. The recovery progress was favorable, obviating the necessity for hospitalization or admission or additional treatment.

## Discussion

The differential diagnosis of acute abdomen in children includes a range of medical and surgical conditions. Physicians are required to conduct a thorough clinical history and physical examination to minimize unnecessary surgical procedures [7]. In an acute surgical abdomen, pain typically precedes vomiting, whereas in medical conditions, vomiting often occurs before the onset of abdominal pain [11]. Abdominal pain in children differs with age, concomitant symptoms, and pain site, and while most cases are self-limiting, there are certain diseases that can be life-threatening such as appendicitis, intussusception, and intestinal obstruction which represent 20% of emergency visits presenting with severe abdominal pain [11]. The most common and prevalent medical cause of abdominal pain in children is acute gastroenteritis which presents with diarrhea, intense cramping, fever, and abdominal pain, and appendicitis is the most common surgical cause [11]. 

In addition to a thorough assessment of the patient's medical history, including symptom onset, progression, location, intensity, precipitating and relieving factors, and associated symptoms, as well as a detailed physical examination involving inspection, palpation, auscultation, and percussion, specific laboratory and imaging evaluations are essential for an accurate diagnosis. It is recommended to perform a complete blood count and a urinalysis in all children presenting with acute abdominal pain. Ultrasound and CT scan are the most frequently utilized imaging studies in the emergency department. Although CT is more specific for identifying the cause of an acute abdomen, ultrasound is recommended as the initial imaging tool for pediatric abdominal pain due to its non-invasive nature, absence of radiation, and lower cost [11].

In both our cases, despite the presentation mimicking a surgical abdomen, immediate surgical pathology was initially ruled out through clinical evaluation, imaging studies, and laboratory assessments. 

When evaluating patients presenting with an acute abdomen, irritable bowel syndrome (IBS) should be considered as a differential diagnosis. IBS can mimic acute abdomen symptoms, which manifest with changes in bowel habits, including abnormal motility and defecation, often associated with abdominal pain, without any discernible underlying structural abnormality. A study conducted in Poland in 2006 demonstrated that EAEC was more prevalent in patients with IBS, with EAEC detected in 81.8% of the cases [12].

A study conducted in Germany in 1997, involving a pediatric population under 16 years of age with a sample size of 798 patients, demonstrated the presence of EAEC in fecal samples using PCR in 16 children, out of which five presented acute abdomen symptoms as their main clinical manifestation [13].

In 1944, strains of *E. coli* capable of invading the colonic mucosa in a manner similar to *Shigella* were identified and termed EIEC, exhibiting significant genetic similarities. Despite the new insights gained into the physiology and pathogenic mechanisms, as well as advancements in antibiotic management, infectious gastroenteritis caused by these bacteria persists as one of the top four causes of diarrheal diseases in children under five years of age in developing countries [14].

Given that both patients presented with intense abdominal pain and lacked other risk factors for *E. coli *infection, initial consideration of such an infection was quite low. The presence of reduced stool consistency played a pivotal role in raising suspicion of an infectious pathology. It is imperative for both clinicians and surgeons to consider the possibility of an infectious etiology in patients presenting with abdominal pain and diminished fecal consistency. This approach is crucial in order to avoid unnecessary surgeries in patients who could benefit from antibiotic treatment. 

Considering that many infections caused by EAEC and EIEC can be asymptomatic or self-limiting, the foundational approach to treatment is oral rehydration and in severe cases intravenous rehydration. Nevertheless, for symptomatic patients, ciprofloxacin 500 mg twice a day for seven days or rifaximin 200-400 mg three times a day for three days can be administered. This therapeutic regimen has demonstrated efficacy in reducing the duration of diarrheal symptoms [7,15].

The widespread use of antibiotics, both prescribed and self-administered, has led to significant antimicrobial resistance among enteropathogens responsible for acute diarrhea. Rifaximin is an antimicrobial agent that can achieve high concentrations in the intestinal lumen [16]. However, in Mexico, the susceptibility of enteropathogenic strains isolated from children remains unknown. In 2017, a study conducted in Mexico compared the in vitro susceptibility of enteropathogenic bacteria isolated from children with acute diarrhea in five states to rifaximin and seven other antibiotics commonly prescribed in the region for infectious gastroenteritis. The results showed that 100% of the studied enteropathogens were susceptible to rifaximin. They exhibited sensitivity above 70% only to ciprofloxacin and fosfomycin, while sensitivity was below 60% for neomycin, trimethoprim/sulfamethoxazole, and ampicillin, with a trend towards lower susceptibility to ampicillin [16].

Considering the importance of determining the etiology in our patients, it is advisable to consider requesting an infectious panel using PCR on stool samples, as this can identify the pathogens responsible for the clinical presentation. Following the initiation of targeted treatment, rapid improvement was observed. While these strains of *E. coli *are well-recognized causes of diarrhea, it's essential to note that they can also lead to abdominal pain, thereby mimicking a presentation of acute abdomen.

## Conclusions

Consideration of infectious etiologies is crucial in the evaluation of patients presenting with abdominal pain suggestive of acute abdomen. It is essential to recognize that pathogens such as EAEC and EIEC have been documented highlighting their potential role as causative agents of abdominal pain that may mimic acute abdomen presentations. By thoroughly exploring these possibilities, healthcare providers can effectively avoid unnecessary surgical interventions, thereby optimizing patient care and outcomes. Furthermore, the implementation of targeted antibiotic therapies directed at the identified pathogens has demonstrated efficacy not only in reducing the duration of hospitalization but also in significantly improving the symptomatic profile of affected patients. This approach enhances patient comfort and facilitates faster recovery while ensuring efficient utilization of healthcare resources. Therefore, a comprehensive approach to identify and treat infectious causes of abdominal pain is pivotal in achieving favorable clinical outcomes.
